# The paradox of sham therapy and placebo effect in osteopathy: A systematic review

**DOI:** 10.1097/MD.0000000000004728

**Published:** 2016-09-02

**Authors:** Francesco Cerritelli, Marco Verzella, Luca Cicchitti, Giandomenico D’Alessandro, Nicola Vanacore

**Affiliations:** a Clinical-based Human Research Department, COME Collaboration, Pescara; b Department of Neuroscience, Imaging and Clinical Sciences; c Institute for Advanced Biomedical Technologies (ITAB), “G. d’Annunzio” University of Chieti-Pescara, Chieti; d Accademia Italiana Osteopatia Tradizionale, Pescara; e National Centre for Epidemiology, Surveillance and Health Promotion, National Institute of Health, Rome, Italy.

**Keywords:** complementary medicine, manual medicine, nocebo, osteopathy, pain, ultrasound

## Abstract

**Background:**

Placebo, defined as “false treatment,” is a common gold-standard method to assess the validity of a therapy both in pharmacological trials and manual medicine research where placebo is also referred to as “sham therapy.” In the medical literature, guidelines have been proposed on how to conduct robust placebo-controlled trials, but mainly in a drug-based scenario. In contrast, there are not precise guidelines on how to conduct a placebo-controlled in manual medicine trials (particularly osteopathy). The aim of the present systematic review was to report how and what type of sham methods, dosage, operator characteristics, and patient types were used in osteopathic clinical trials and, eventually, assess sham clinical effectiveness.

**Methods:**

A systematic Cochrane-based review was conducted by analyzing the osteopathic trials that used both manual and nonmanual placebo control. Searches were conducted on 8 databases from journal inception to December 2015 using a pragmatic literature search approach. Two independent reviewers conducted the study selection and data extraction for each study. The risk of bias was evaluated according to the Cochrane methods.

**Results:**

A total of 64 studies were eligible for analysis collecting a total of 5024 participants. More than half (43 studies) used a manual placebo; 9 studies used a nonmanual placebo; and 12 studies used both manual and nonmanual placebo. Data showed lack of reporting sham therapy information across studies. Risk of bias analysis demonstrated a high risk of bias for allocation, blinding of personnel and participants, selective, and other bias. To explore the clinical effects of sham therapies used, a quantitative analysis was planned. However, due to the high heterogeneity of sham approaches used no further analyses were performed.

**Conclusion:**

High heterogeneity regarding placebo used between studies, lack of reporting information on placebo methods and within-study variability between sham and real treatment procedures suggest prudence in reading and interpreting study findings in manual osteopathic randomized controlled trials (RCTs). Efforts must be made to promote guidelines to design the most reliable placebo for manual RCTs as a means of increasing the internal validity and improve external validity of findings.

## Abstract 2

Supplemental Digital Content is available in the text

## 1 Introduction

Placebo is defined as false treatment or false therapy and its effects are a well-recognized phenomenon in medicine.^[1]^ Although an inert physically or pharmacologically therapy is administered,^[2]^ placebos can produce improvements in patients’ symptomatology.^[3]^ A robust body of literature clearly demonstrates the neurobiological correlates of placebo and placebo response in both healthy subjects and patients.^[4–6]^ Placebo (and the related nocebo) effects seemed to be influenced by several psychosocial factors including patient expectation, patient–doctor relationship, and therapeutic rituals.^[5,7]^ These factors seem to elicit neuropsychological mechanisms like conditioning,^[1,8–10]^ expectation,^[11–13]^ and reward,^[8]^ which in turn drive modifications in the brain–body interactions^[8]^ through endocrine,^[2,10]^ immune,^[8]^ and autonomic^[2,8]^ systems.

Placebo has to be carefully considered in trial design^[8,14]^ as it can mask the real effects of a given pharmacological or nonpharmacological treatment.^[8]^ For this reason, placebos are considered fundamental control groups in randomized controlled trials (RCTs).^[14,15]^ Critically, placebos have been tested and used in double blinded clinical trials investigating drug-based therapy effects. Using this study design, by definition, the therapist/drug administrator has to be blinded to the treatment. However, when applying this methodology to other health care areas, the scenario might be unsuitable. That is the case of manual complementary and alternative medicines, including osteopathy, where the use of placebos is inherently biased by the therapist, who actively administers the manual treatment.^[16,17]^ In fact, these “drug-driven” scientific and methodological standards might have produced important consequences about the improper interpretation of studies in manual treatment with potential misinterpretation of its effects from a public health perspective.^[18–20]^ Furthermore, the literature reports guidelines and recommendations to conduct nonpharmacological trials,^[21]^ but there is still lack of a common and robust manual placebo paradigm.^[22]^ Expected advantages from creating a paradigm will include an improvement of placebo awareness not only in the “core clinical practice curriculum of all health practitioners,”^[1]^ but also in the field of research.

In the last decades, osteopathic clinical trials have been steadily increasing in number and type, addressing various diseases and patient populations with different methodologies. Considering comparison groups, osteopathic research often used the so-called “false treatment” as control arm.^[23–27]^ This type of treatment can be referred to as sham therapy or placebo; however, its use and methodology are left to researchers’ discretion rather than to formal guidelines and/or recommendations. There is a need to clarify and codify the sham methods used in manual therapy research to assess and develop a robust paradigm that may become the standard placebo arm of a trial. Therefore, with the purpose to assess the use of sham therapy in manual medicine, primarily osteopathy, the aim of this systematic review was to describe the methods used for placebo/sham therapy defining similarities and diversities among osteopathic studies about different aspects: methodology, dosage, operator's characteristics, and type of patients. In addition, the clinical effectiveness of sham procedures was explored comparing control and intervention groups.

## 2 Methods

This systematic review included single- and multicenter RCT, quasi-RCT, controlled clinical trials, interrupted time series, and controlled before and after studies. Observational studies, cohort studies, cross-sectional studies, case-control, case-series, and case-report studies as well as abstract and animal studies were excluded. Study reports must have been written in English. Research including patients with any medical condition as well as healthy (asymptomatic) subjects of either sex and any age were considered eligible.

In addition, included studies had to have at least a control group in which a form of sham therapy was provided. Interventions and sham therapy could be applied alone or in addition to conventional treatments (i.e., pharmacological cointerventions, counseling, or advice prescription).

### 2.1 Data sources and searches

The identification of the studies was conducted by a comprehensive computerized search of MEDLINE (http://www.ncbi.nlm.nih.gov/pubmed), Scholar google (http://scholar.google.it), SCOPUS (http://www.elsevier.com/onlinetools/scopus), clinicaltrials.gov, chiloras/MANTIS, OSTMED.DR (http://ostmed-dr.com/), Osteopathic Research Web (http://www.osteopathic-research.com/), and the Cochrane Library (http://www.thecochranelibrary.com). Other sources included gray literature, national trials registers, web searching, and conference proceedings. Search terms are included in the Supplemental Content 1 (see Table, Supplemental Content, that reported the search strategy used). The search was conducted from journal inception to December 2015. Duplicate records were identified in EndNote and eliminated.

### 2.2 Study selection

Two reviewers (MV and LC) with expertise in the field of osteopathic medicine research independently conducted the study selection based on the explicit search strategy. Discrepancies were resolved by consensus with FC as an arbiter. There were discussions about 5 studies that were ultimately excluded. According to inclusion criteria, the reviewers independently screened titles and abstracts and full-text were retrieved and assessed.

### 2.3 Data extraction and quality assessment

Data extraction was performed independently by the 2 reviewers, in terms of population characteristics, type of interventions, type of sham, study results, and all the other descriptive characteristics of the included trials (i.e., nationality, year of publication). All disagreements were discussed and resolved by consensus. If data were not reported in the study, the author was contacted. The authors were emailed twice with the aim to obtain complete data. All analyzed data were stored in a dedicated hard disk, accessible only by the 2 reviewers.

Each study was independently evaluated. According to the Cochrane methods, the risk of bias was categorized in low, high, and unclear across the following domains: Sequence Generation; Allocation Concealment; Blinding to Personnel; Blinding to Outcome Analysis; and other bias.^[18]^ Reviewers collected the quality assessment of reports of RCTs in terms of Jadad scores, considering the description and sequence of randomization, the double blind procedure, its appropriateness, and the description of withdrawals and dropouts (range 0–5).^[28]^

### 2.4 Data synthesis and analysis

Sham therapy was categorized into 3 groups: manual, nonmanual, and combined. The aim was to classify control groups based on the delivering of manual or non-manual manipulation. Therefore, the manual sham group was defined as studies delivering manual manipulations only; the nonmanual sham group was established as applying any type of control intervention without manual contact; and the combined sham group was represented by research using both manual and nonmanual procedures. For each study, the number of subjects receiving any form of sham therapy was computed. In addition, sham operators and type of sham were detailed and analyzed. The counts of the separate studies were summed to obtain the cumulative number of subjects/operators reporting each type of sham/therapy administered. The final counts were used to combine data from individual studies. Data were reported as mean, point estimate, percentage, and range. Dispersion was presented as standard deviation and 95% confidence interval (CI). For continuous data, mean differences with 95% CIs were used. For dichotomous outcomes, to compare the different classes of placebos for each given category, the observed total number of subjects were entered into 2 × 2 contingency table and results were presented as odds ratio (OR) with 95% CI. Nonoverlapping CIs of the rates of placebo groups indicate significant differences among the different categories. Moreover, to study the various type of sham therapies a conservative approach was chosen to analyze data. Therefore, χ^2^ test, Student *t* test, and ANOVA with Tukey post hoc analysis were used to compare groups. To control for multiple tests, a Bonferroni correction of α value was adopted. Furthermore, the Yates’ correction for continuity was used, required in one-degree-of-freedom situations.^[29]^ Statistical significance was set at α < 0.05.

Other variables such as sample size, sex, age, weight, healthy subjects, withdrawals, and publication year were also considered in the analysis.

To explore the possibility to run a meta-analysis, studies were not pooled if there was significant heterogeneity. A stepwise heterogeneity assessment was performed. Clinical and methodological heterogeneity were assessed first considering the following levels: patients, intervention, outcome, control event rate/baseline risk, research setting, comparison conditions, early stopping rules, and population risk.^[30]^ Statistical heterogeneity was assessed using the I^2^ statistic, only if clinical and methodological homogeneity were satisfied. The software used for statistical analyses was R v 3.2.0.

## 3 Results

### 3.1 Description of studies

**Figure 1:**
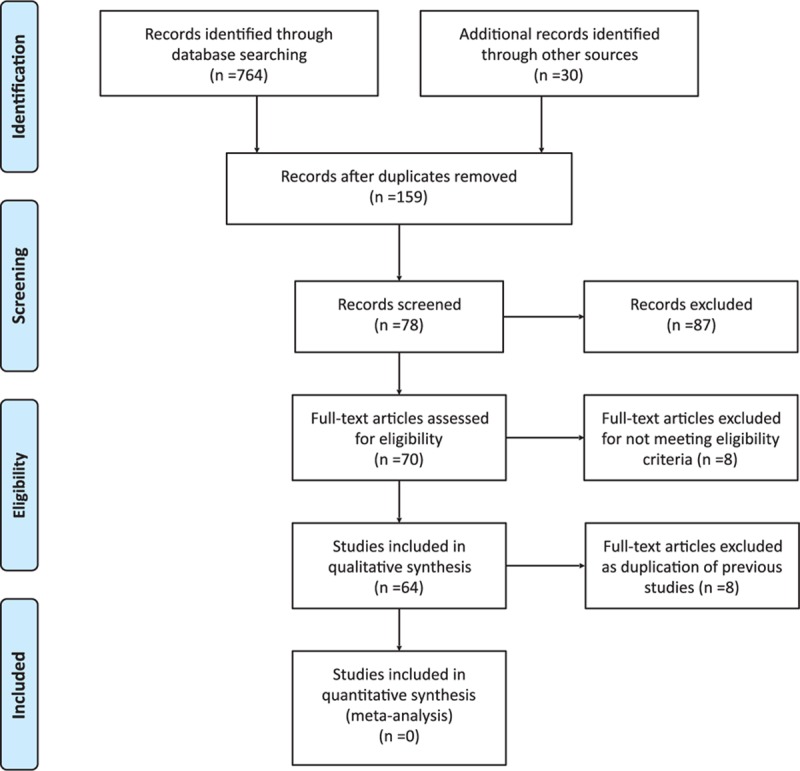
Flow diagram of the study selection.

Seventy full-text studies were assessed for eligibility (Fig. 1). Data and publications emanating from the same study were considered duplicates and therefore excluded. The final sample was 64 studies enrolling 5024 patients. Characteristics of the 3 placebo arms are shown in Table 1.

**Table 1:**
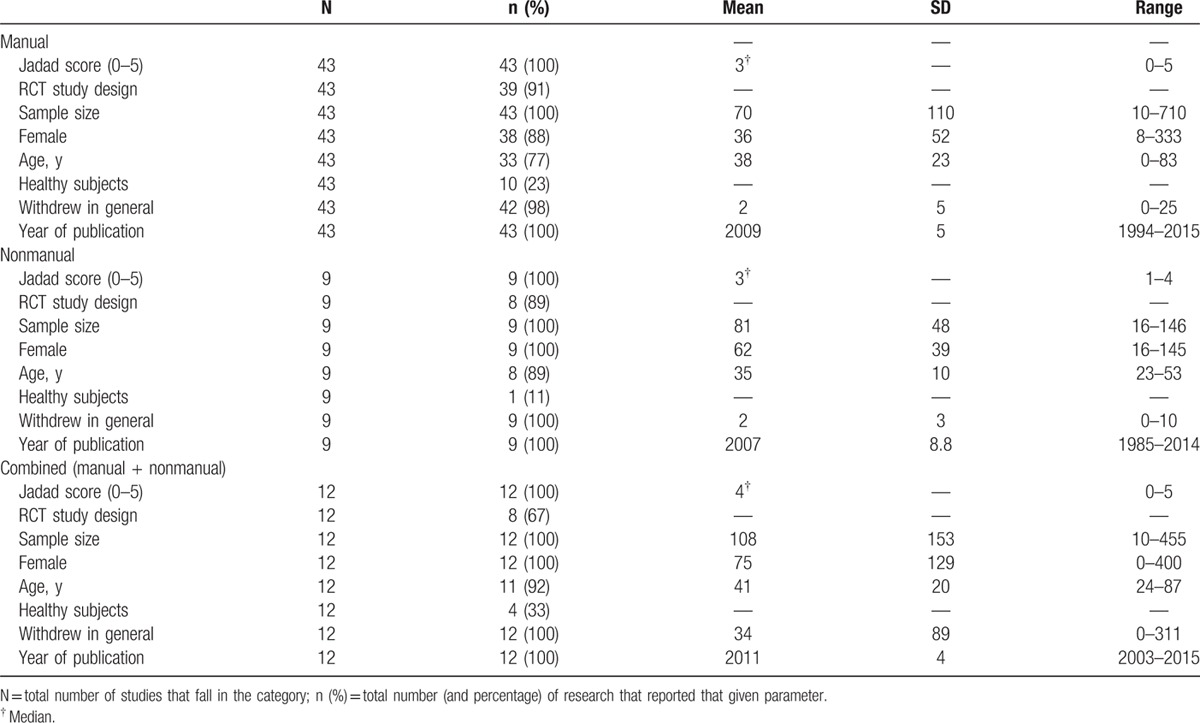
Characteristics of the sham groups, divided into the manual, nonmanual, and combined (manual and nonmanual).

Descriptively, the majority of research was published between 2000 and 2015 (n = 61; 95%), mainly in the United States (n = 26; 41%) and Europe (n = 28; 44%), designed as RCT (n = 58; 91%) with 2 study arms (n = 40; 63%) and within the field of musculoskeletal care (n = 30; 47%).

### 3.2 Clinical comparison between the 3 placebo categories

Analyses revealed statistically significant differences between the 3 groups in terms of number of studies (χ^2^ = 40.55; *P* < 0.001). Marginal differences were shown regarding the type of study design used, with a larger number of RCTs in manual and nonmanual compared with combined sham group (χ^2^ = 4.56, *P* = .10). The number of withdrew subjects, that is the number of subjects who withdrew from the type of study that is reported (manual, nonmanual, combined), showed significant differences (*F*_(2,54)_ = 3.331; *P* = .04). Post hoc comparison showed significantly more subjects dropped out in the combined placebo group than in either other group (−31.76; 95% CI −61.91, −1.61; *P* = 0.03).

No significant differences in sample size, age, Jadad score, number of healthy subjects and females enrolled, and years of publication were observed (Table 1).

Table 2 shows the number and percentage of sham characteristics across the different placebo groups. The rates of type of touch varied widely across trials and placebos. Light touch was the most used approach, particularly in the combined group. The latter showed a statistically significant percentage difference compared with the manual sham group (χ^2^ = 3.50, *P* = 0.06, Z = −2.26, *P* = 0.02). Nonmanual placebo used mainly ultrasound, whereas combined sham therapy preferred usual care or no intervention controls.

**Table 2:**
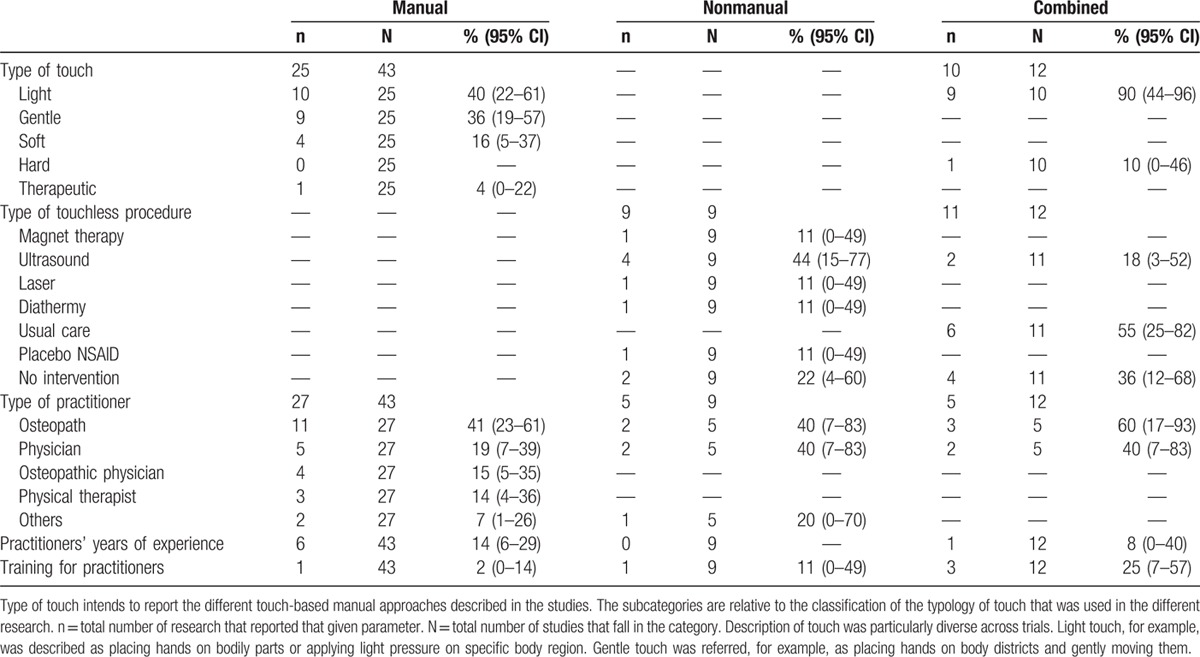
Different characteristics of sham scenario in manual, nonmanual, and combined groups.

With regard to the type of practitioner who administered the sham therapy, osteopaths were the most prevalent group with higher percentage in the combined group. Years of experience were reported in few studies (7/64; 11%); however, considering the number of years of practitioners’ experience, data showed a significant difference between the 3 groups (*F*_(2,50)_ = 6.75; *P* = 0.048). Tukey post hoc comparison demonstrated that manual sham group operators were older than those in the combined trials (9.67; 0.10, 19.23; *P* = 0.05). Only 5 studies (9%) described whether practitioners received consensus training before the commencement of the research, with 7 trials (8%) declaring the absence of preconsensus training. Moreover, in >50% of trials lack of comparability was showed between sham therapy and real intervention.

### 3.3 Differences in missing data reporting and sectorial publication

Data showed lack of reporting sham therapy information across studies. Dosage, number of session performed, period of sham treatment, type of procedures applied (protocol-based or personalized), and type of sham approach used were significantly underreported. Less than 30% of all included studies described at least 3 of the above-mentioned aspects.

On average, the manual sham group trials reported information on the 27.8% (range 2%–55%), nonmanual on 41.5% (0%–100%), and combined 83.3% (9%–100%) with a statistical significant difference between groups (χ^2^ = 7.38, *P* = 0.02). Specifically, ORs between placebo categories showed the followings: manual versus nonmanual (0.66; 0.32–1.40; *P* = 0.28), manual versus combined (0.43; 0.23–0.80; *P* < 0.01), and nonmanual versus combined (0.64; 0.27–1.50; *P* = 0.30). Thus, the probability of not reporting information is significantly higher in the manual trials than all other placebo groups.

Taking into account the probability of publishing on sectorial (osteopathic) journals, χ^2^ test showed a marginal difference between the 3 categories (χ^2^ = 4.96, *P* = 0.08). In fact, manual placebo trials were more likely to be published in osteopathic journals compared with the other categories (manual: 20/43, 47%; nonmanual: 1/9, 11%; combined: 3/12, 25%).

### 3.4 Type of placebo used by age group

**Figure 2:**
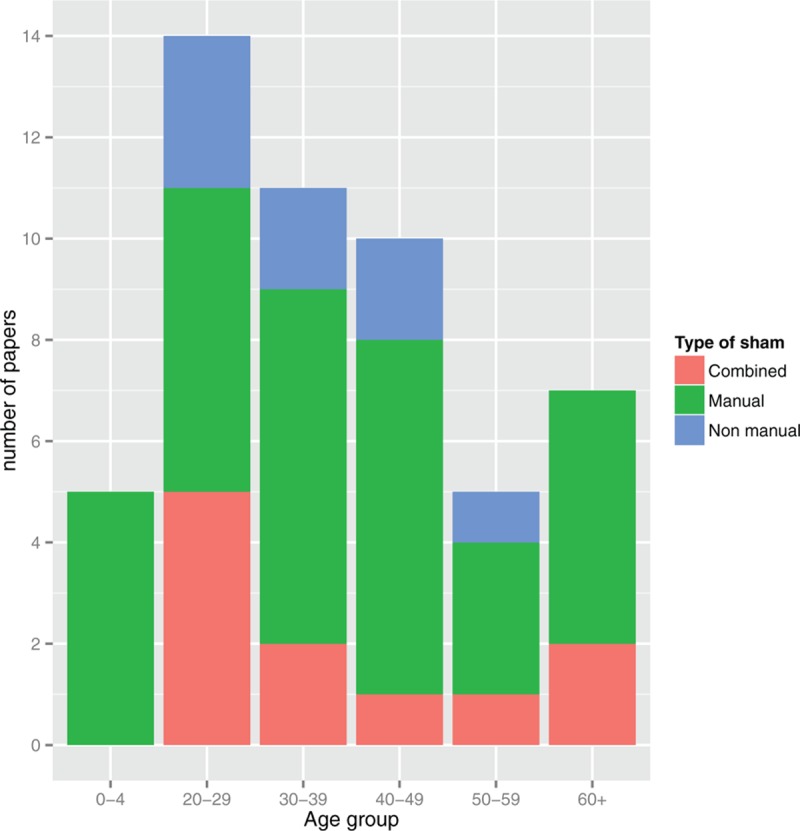
Number of papers included by type of sham and age group.

Considering the type of placebo according to age group, it was shown that there might be an age-related use (Fig. 2). All the 3 approaches were used in the adulthood (between 20 and 50 years old). Manual placebo was the only approach used in babies, whereas in the elderly only manual and combined were applied. A further analysis showed no statistically significant association between age group and type of placebo (χ^2^ = 5.02, *P* = 0.29).

### 3.5 Adverse events across studies

Only 23% of research reported data on adverse events of which manual sham group showed the highest reporting percentage (12/43; 28%; range 16%–44%) compared with nonmanual (1/9; 6%; 0%–43%) and combined (2/12; 17%; 3%–49%), although not statistically significant (χ^2^ = 1.55, *P* = 0.46). As far as ORs are considered, data showed that the likelihood of reporting adverse events is larger in the manual sham group than both the combined group (1.67; 0.33–8.53; *P* = 0.54) and nonmanual sham trials (2.51; 0.29–21.84; *P* = 0.40). However, the combined category had higher probability than nonmanual sham group (1.50; 0.12–19.24; *P* = 0.76).

Among the 14 trials describing adverse events, 10 (71%) reported absence of symptoms whereas 5 showed mild side effects from the sham therapy applied. Furthermore, among a total of 720 reporting patients, 42 complained having adverse events (5.9%).

### 3.6 Sample size calculation

Only the 31% (20/64) of research preestablished a sample size considering statistical and clinical parameters. The vast majority of studies did not report any information or declare to not having calculated the sample size. Among the 3 placebo categories, the combined was the group with the highest percentage of trials with sample size calculation reporting (6/12; 50%). In manual and nonmanual sham groups, sample size calculation was reported in the 28% and 22%, respectively. No statistically significant difference was shown (χ^2^ = 2.53, *P* = 0.28).

### 3.7 Assessment of homogeneity across placebos groups

No research was considered acceptable for meta-analyses as did not satisfy inclusion criteria. Specifically, none of the studies used similar sham therapies, leading to high (clinical) heterogeneity of sham approach used, therefore preventing any further quantitative analysis. This limited, also, the possibility to compute statistical homogeneity through I^2^, and precluded additional pooling for exploring any clinical effectiveness of sham procedure used. Supplemental Content 2 (see Table, Supplemental Content, that reported information regarding eligible research) showed details regarding included studies eligible for meta-analysis. Supplemental Content 3 (see Table, Supplemental Content, that showed details on sham procedures) reported information on manual and nonmanual sham maneuvers used by trials.

### 3.8 Risk of bias evaluation

Reliability between reviewers was estimated. Kappa score was equal to 0.793 for quality assessment in terms of Jadad scores, whereas κ = 0.801 for risk of bias assessment. Therefore, reliability was considered high between reviewers. Out of 64 studies^[31–92]^ included in the current systematic review, 9 were not RCTs.^[31–39]^ The remaining 55 RCTs were evaluated in the different categories of bias.

The majority of the trials included presented a low risk of bias for sequence generation and allocation concealment. More than half of the studies claimed that the research personnel and/or patients involved in their respective trial were not aware of study design and outcomes, but did not report any information regarding blinding of outcome assessors. None of the RCT reported their protocols, thus the assessment for selection bias was not possible to perform. The quality of included studies was further assessed considering the followings: informed consent, conflict and declaration of interest, reporting funding source, ethical approval, confidentiality, access to data, trial registration, data collection, data management, and data monitor committee. A large number of the research did not describe sufficient information regarding the majority of the categories considered (see Table, Supplemental Content 4, that reported information regarding risk of bias).

## 4 Discussion

The results of the present review could be summarized as follows: high (clinical) heterogeneity regarding placebo/sham used between studies, lack of reporting information on placebo/sham methods, and within-study variability between sham and real treatment procedures.

First, interstudy clinical heterogeneity was significantly large; indeed, several types of sham therapies were reported. Some studies used a manual sham approach, whereas others used nonmanual contact or combined manual and nonmanual procedures. Interestingly, high variability within each category was revealed. In fact, several different types of touch were applied in manual sham studies. Consistently, many diverse instruments/physiotherapeutic tools were used in nonmanual sham trials. In addition, placebo pills and/or no intervention were used in other research. This scenario resulted in high clinical heterogeneity in sham manual procedures applied, which prevented a meta-analysis, although studies were homogeneous for other aspects (i.e., clinical field, intervention, participants). It can be argued that this great diversification in the control group could produce low internal and external validity of results. Therefore, lack of replicability could be a consequence of this methodological bias. These aspects might lead studies to decrease the likelihood of being compared and, thus, affecting the clinical validity.

Second, the analysis of missing data showed a consistent, structured, and significant lack of reporting information about sham procedure. In particular, trials systematically underreported details regarding the following aspects: sham dosage (i.e., duration, session, period); sham procedures (i.e., protocol-based or personalized, type of approach); and sham operator (type of practitioner, number of operators, operator's years of experience, training for practitioner, operator background, supervision/tutoring). This context might produce a reduction of robustness of placebo groups, increasing significantly the likelihood of having skewed results, high risk of bias (i.e., tools’ reliability and validity, performance bias), incomplete data reporting and, thus, reduced external validity of findings.

Third, sham treatment, by definition, should resemble the active treatment in any aspect except for technique. Using simplistically the placebo-drug paradigm, it can be argued that the real effect of “active ingredient” in osteopathic manipulative treatment (OMT) is equal to the effects of real OMT minus the effects of placebo/sham OMT. This equation might suggest that technique applied should be the only deterrent factor between real and placebo OMT. However, the results from the present review highlight that inconsistent procedures were applied between study and control groups. This might increase the probability of having unmatched groups and, therefore, change any estimation of OMT effect.

Furthermore, it can be claimed that, despite the effectiveness of a procedure, other “confounding” factors could be associated to results. Applying different touch strategies was argued to influence clinical outcomes. Noteworthy, light touch^[93]^ is known to activate low-threshold mechanoreceptors through the so-called C-tactile fibers. This was demonstrated to modify the autonomic nervous system functions^[94,95]^ (see McGlone 2014 for review).^[96]^ Number of sessions, operators’ characteristics, and preconsensus training could all potentially interact with study outcomes. Therefore, several additional factors need to be considered to rate the quality of studies and estimate intervention effectiveness. From a clinical point of view, the assessment of these components is thus crucial to correctly evaluate the validity and reliability of research.

Interestingly, although the quality of studies was generally moderate, high risk of bias was showed in all items concerning the description of intervention, thus performance bias. This will possibly imply that patients did not receive a similar amount of attention, thus influencing the final outcome.

In general, the use of placebo is a key tool in any RCT.^[14,15,97]^ It has been suggested that it is essential to shed light on the real effect of a given treatment taking into account several potential psychosocial confounders.^[8]^ Methods of placebo were historically linked to pharmacological trials where the “sugar pill” was considered the placebo par excellence. In manual sham RCTs, it could, however, be very complex to isolate the “active ingredient” for several reasons.^[16]^ In fact, as demonstrated by this review, the different “sham therapies” were significantly heterogeneous according to different aspects (population, operator, real treatment-like procedures, osteopathic techniques) and context (i.e., clinical). Moreover, after evaluating the quality and type of reporting information, osteopathic trialists lack to report key details on placebo and control group. For example, on the one hand Ruffini et al conducted a well-designed RCT including sufficient details of placebo procedures to affirm that OMT might have real parasympathetic and trophotropic effects.^[26]^ This implies that findings could be considered valid and reliable at the light of sufficient methodological details and quality. On the other hand, Barnes et al carried out an RCT suggesting that OMT could be relevant in the improvement of cvical mobility.^[41]^ Although the positive results described, the poor methodology as well as the insufficient information provided by the authors might question on the credibility and robustness of data.

Therefore, it can be clear that the above-mentioned research, although clinically positive, might produce completely different study assessments and effects of osteopathic intervention.

Moreover, published guidelines suggest ways to reduce risk of bias.^[98,99]^ However, the concept of bias was itself built on pharmacological trials. It was difficult for nonpharmacological research to resist the compelling argument for the use of sham therapy demonstrated by pharmacological trials. As a consequence, this exposes the drug-free study designs to several methodological flaws, biases, for example, performance bias as not respecting the blinding of personnel,^[21]^ and, thus, misinterpretation of quality and results. As a matter of fact, the present review highlighted this “paradox” of sham therapies calling for pragmatic methodological solutions. Practical recommendations for further studies included detailed description of sham procedures used, focusing on dosage, frequency, number of sessions, period of sham treatment, type of procedure, and approach applied. In case of manual sham treatment, description of type of manual approach used with details is required. Attention should be paid to the use of sham arms that mimic and are similar to the real intervention. Practitioners’ selection and consistency are another relevant aspects to be considered when planning an appropriate placebo-controlled RCT.

Collectively, results from the present systematic review suggest prudence in reading and interpreting study findings in manual osteopathic RCTs; the necessity to consider sham group as relevant part of osteopathic manual medicine trials; to accurate assess and plan sham procedures in RCTs; and the need to shift from a pharmacological research-based scenario to a multidisciplinary manual-based research guidance.

Several limitations could be pointed out in the present systematic review. There could be some potential sources of publication bias.^[100]^ Although an attempt was made to identify unpublished research, which is more likely to have negative outcomes,^[101,102]^ the search strategy may have left out relevant studies not currently indexed. Moreover, limiting the search to publications written in English could have skewed the general results.^[103]^

## 5 Conclusions

The aim of the present review was to explore the extent to which sham therapies were used in manual medicine, in particularly in osteopathic clinical trials. High heterogeneity regarding placebo used between studies, lack of reporting information on placebo methods and within-study variability between sham and real treatment procedures suggest prudence in reading and interpreting study findings in manual osteopathic RCTs. Efforts must be done to promote guidelines to design the most reliable “sugar pill” for manual RCT. Robust recommendations should be based on methodological aspects and on neurobiology of placebo. This will facilitate structured versus spontaneous sham therapy reporting. Therefore, effects are predicted on study validity and between-study homogeneity. Arguably, a valid, reliable, reasonable, and common placebo will increase the internal validity and improve external validity of findings.

## Acknowledgments

The authors thank Dr Jorge Esteves for his help in critically reviewing the paper.

## Supplementary Material

Supplemental Digital Content
