# Supplementary material for: The paradox of sham therapy and placebo effect in osteopathy: A systematic review
Source: Medicine (Baltimore). 2016 Sep 2;95(35):e4728. doi: 10.1097/MD.0000000000004728 (PMC5008597; doi:10.1097/MD.0000000000004728)
Supplement: Supplemental Digital Content [file medi-95-e4728-s001.doc]

Search strategy

osteopat* manipulat* treatment (MeSH terms), osteopat* therapy (All terms), cranial osteopat*, manipulative (All terms), cranial osteopat*, osteopathic medicine (MeSH terms), Craniosacral, Cranial sacral treat*, sham therapy (all terms), placebo effect (MeSH terms), sham treatment (MeSH terms), sham therapy AND osteopat*, sham therapy AND osteopat* therapy, sham therapy AND osteopat* manipulat* treatment, sham therapy AND cranial osteopat* manipulative, sham therapy AND cranial osteopat*, sham therapy AND osteopathic medicine, sham therapy AND craniosacral, sham therapy AND Cranial sacral treat, placebo effect AND osteopat*, placebo effect AND osteopat* therapy, placebo effect AND osteopat* manipulat* treatment, placebo effect AND cranial osteopat* manipulative, placebo effect AND cranial osteopat*, placebo effect AND osteopathic medicine, placebo effect AND craniosacral, placebo effect AND Cranial sacral treat, sham treatment AND osteopat*, sham treatment AND osteopat* therapy sham treatment AND osteopat* manipulat* treatment, sham treatment AND cranial osteopat* manipulative, sham treatment AND cranial osteopat*, sham treatment AND osteopathic medicine, sham treatment AND craniosacral, sham treatment AND Cranial sacral treat*, osteopathic manipulative treatment "sham therapy", osteopathy "sham therapy", "sham therapy" osteopat* therapy, cranial osteopathy manipulative "sham therapy", osteopathic medicine "sham therapy", Craniosacral "sham therapy", cranial osteopath* "sham therapy", Cranial sacral treat* "sham therapy”, cranial osteopat* manipulative "sham therapy", osteopathic manipulative treatment "placebo effect", osteopathy "placebo effect", osteopat* therapy "placebo effect", cranial osteopathy manipulative "placebo effect", osteopathic medicine "placebo effect", Craniosacral "placebo effect", cranial osteopath* "placebo effect", Cranial sacral treat* "placebo effect", cranial osteopat* manipulative "placebo effect", osteopathic manipulative treatment "sham treatment", osteopathy "sham treatment", osteopat* therapy "sham treatment", cranial osteopathy manipulative "sham treatment", osteopathic medicine "sham treatment", Craniosacral "sham treatment", cranial osteopath* "sham treatment", Cranial sacral treat* "sham treatment", cranial osteopat* manipulative "sham treatment".

**Appendix table 2**

**Summary of quantitative meta-analysis sorted by outcome of interest**

The document summaries information after assessing studies suitable for meta-analysis. This allows the reader to have a clear idea regarding trials included in the present systematic review organized by topic – outcome of interest – and the likelihood of performing a meta-analysis based on data from selected research.

The structure of reporting is organized as follows:

- Topic – outcome of interest
- Population of interest
- List of studies included by outcome of interest
- *Common measures*: (detailing the measurements in common between studies)
- *Sham:* (manual, non-manual, mixed)
- ***Likelihood of meta-analysis:* Y / N / Unclear**
- *Reasons*: if N from Likelihood of meta-analysis
- *Concerns*: if Unclear from Likelihood of meta-analysis. Solution used was detailed

**Autonomic Nervous System**

| **Study (year)** | **Outcome** | **Type of population (mean age ± sd)** |
| --- | --- | --- |
| Ruffini (2015) | Heart rate variability measures | Healthy adult (26±8) |
| Giles (2013) | Heart rate variability measures | Healthy adult (25±2) |
| Henley (2008) | Heart rate variability measures | Adult (NA) |

- *Population: healthy adults
  Common measures:* LF, HF, LF/HF ratio
- *Sham:* manual
- ***Likelihood of meta-analysis:* Unclear**
- *Concerns:* Giles used LF, HF and LF/HF power (not clear if absolute power or normalized units was reported) whereas Henley used normalized unit. Authors were contacted, but no answers were received.

**CNS and PNS**

Population: adults

| **Study (year)** | **Outcome** | **Type of population (mean age ± sd)** |
| --- | --- | --- |
| Howell (2006) | H-reflex | Adults with achilles tendinitis (37 ±NA) |
| Wynne (2006) | H-reflex changes | Adults with plantar fascitis (range 20-66) |

- *Common measures:* H and F waves
- *Sham:* manual
- ***Likelihood of meta-analysis:* N**
- *Reasons*: studies did not use the same stimulation method as well as the same area of stimulation

**Disability - RMDQ**

Population: adult with non specific low back pain

| **Study (year)** | **Outcome** | **Type of population (mean age ± sd)** |
| --- | --- | --- |
| Licciardone (2003) | Disability measured with RMDQ | Adults with NSLBP (range 21-69) |
| Licciardone, Kearn, Minotti (2013) | Disability measured with RMDQ | Adults with NSLBP (range 21-69) |
| Licciardone, Minotti (2013) | Disability measured with RMDQ | Adults with NSLBP (range 21-69) |
| Licciardone, Aryal (2014) | Disability measured with RMDQ | Adults with NSLBP (range 21-69) |

- *Common measures:* Roland-Morris Disability Questionnaire
- *Sham:* mixed (manual – Licciardone 2003; ultrasound Licciardone 2013)
- ***Likelihood of meta-analysis:* N**
- *Reasons*: different sham therapies used

General comments: Licciardone, Kearns 2013 and Licciardone, Aryal 2014 used data from Licciardone, Minotti 2013.

**Inactivity**

Population: adult with non specific low back pain

| **Study (year)** | **Outcome** | **Type of population (mean age ± sd)** |
| --- | --- | --- |
| Licciardone (2003) | Inactivity measured by the number of lost work or school days within the preceding 4 weeks | Adults with NSLBP (range 21-69) |
| Licciardone, Minotti (2013) | Inactivity measured by the number of lost work days in the past 4 weeks because of low back pain | Adults with NSLBP (range 21-69) |

- *Common measures:* lost work days
- *Sham:* mixed (manual – Licciardone 2003; ultrasound was additionally used in Licciardone 2013)
- ***Likelihood of meta-analysis:* N**
- Reasons: different sham therapies

**Pain – VAS 100**

Population: adult with (non specific) low back pain

| **Study (year)** | **Outcome** | **Type of population (mean age ± sd)** |
| --- | --- | --- |
| Gibson (1985) | Pain measured by VAS 100 mm | Adults with LBP (36 ± 15) |
| Licciardone, Minotti (2013) |  | Adults with NSLBP (range 21-69) |
| Licciardone, Aryal (2014) |  | Adults with NSLBP (range 21-69) |

- *Common measures:* VAS
- *Sham:* mixed (manual in Gibson 1985, ultrasound in Licciardone, Minotti 2013)
- ***Likelihood of meta-analysis:* N**
- *Reasons*: using of different samples, different assessment time-periods

**Pain – Pressure threshold**

Population: adult with back pain

| **Study (year)** | **Outcome** | **Type of population (mean age ± sd)** |
| --- | --- | --- |
| Degenhardt (2014) | Pain using pressure pain thresholds | Adult with NSLBP (range 20-60) |
| Hamilton (2007) | Pain using pressure pain thresholds | Adult with neck pain (25±5) |
| Mansilla-Ferragut (2009) | Pain using pressure pain thresholds | Adult with neck pain (35±8) |

- *Common measures:* algometer
- *Sham:* mixed (manual - ultrasound)
- ***Likelihood of meta-analysis:* N**
- *Reasons*: different bodily areas of assessing pain (sphenoid- Mansilla; C1 – Hamilton; Lumbar- Degenhardt)

**Pain – IMMPACT**

Population: adult with non specific low back pain

| **Study (year)** | **Outcome** | **Type of population (mean age ± sd)** |
| --- | --- | --- |
| Licciardone, Kearn, Minotti (2013) | Pain using IMMPACT scale | Adults with NSLBP (range 21-69) |
| Licciardone, Minotti (2013) | Pain using IMMPACT scale | Adults with NSLBP (range 21-69) |

- *Common measures:* IMMPACT
- *Sham:* mixed
- ***Likelihood of meta-analysis:* N**
- *Reasons*: data came from the same study (Licciardone, Minotti 2013)

**Quality of life – SF-36**

Population: adult with non specific low back pain

| **Study (year)** | **Outcome** | **Type of population (mean age ± sd)** |
| --- | --- | --- |
| Licciardone (2003) | Quality of life using SF-36 | Adults with NSLBP (range 21-69) |
| Licciardone, Kearn, Minotti (2013) | Quality of life using SF-36 - GH subdomain | Adults with NSLBP (range 21-69) |
| Licciardone, Minotti (2013) | Quality of life using SF-36 - GH subdomain | Adults with NSLBP (range 21-69) |
| Licciardone, Aryal (2014) | Quality of life using SF-36 - GH subdomain | Adults with NSLBP (range 21-69) |

- *Common measures:* SF-36 (GH subdomain)
- *Sham:* mixed (manual – Licciardone 2003; ultrasound was additionally used in Licciardone 2013)
- ***Likelihood of meta-analysis:* N**
- *Reasons*: different sham therapies used. Moreover Licciardone, Keans 2013 and Licciardone Aryal 2014 presented data from Licciardone, Minotti 2013

**Quality of life - depression**

Population: adult with irritable bowel syndrome (IBS)

| **Study (year)** | **Outcome** | **Type of population (mean age ± sd)** |
| --- | --- | --- |
| Florance (2012) | depression | Adult with IBS (47±17) |
| Attali (2013) | depression | Adult with IBS (50±2) |

- *Common measures:* Attali - two-question case-finding instrument; Florance - 13-item BDI
- *Sham:* manual
- ***Likelihood of meta-analysis:* N**
- *Reasons*: different tools for assessing depression

**Quality of life – RMDQ**

Population: pregnant women

| **Study (year)** | **Outcome** | **Type of population (mean age ± sd)** |
| --- | --- | --- |
| Hensel (2015) | Disability measured with RMDQ | Pregnant adults (range 18-35) |
| Licciardone (2010) | Disability measured with RMDQ | Pregnant adults (NA) |
| Licciardone, Aryal (2013) | Disability measured with RMDQ | Pregnant adults (NA) |

- *Common measures:* Roland-Morris Disability Questionnaire
- *Sham:* Mixed (sham Ultrasound + manual contact) and (sham ultrasound)
- ***Likelihood of meta-analysis:* N**
- *Reasons*: Hensel 2015 and Licciardone 2010 used the same outcome and design, but not exactely the same sham therapy. Licciardone 2013 is excluded as presenting data from Licciardone 2010.

Appendix 3. General information regarding sham and control procedures used by each included study

| **Paper** | **Type of placebo** | **Type of touch** | **Description of sham and control** | **Time of session** | **Treatment period** |
| --- | --- | --- | --- | --- | --- |
| 43 | M | NA | Sham: overlapped hands touching the occipital bone and provided random slight non-therapeutic touch of random duration until the end of the session time. | 1 time for 10 min | SS |
| 26 | C | NA | Sham: hands on a sequence of pre-established anatomical areas for 2 min each: right ankle, left knee, right hip, diaphragm, right shoulder, neck, cranium. | 1 time/wk for 25 min | 1 wk |
|  |  | NT | Time control: no intervention | 1 time/wk |  |
| 76 | M | L | Sham: light touch on standardized anatomic areas mimicking craniosacral therapy. Body awareness instructions were given to simulate CST dialog techniques. | 1 time/wk for 45 min | 8 wk |
| 80 | M | G | Osteopathic evaluation + usual care: pre-established osteopathic light-touch structural examination. | 2 times/wk for 20 min | UD |
| 82 | M | G | Osteopathic evaluation + usual care: pre-established osteopathic light-touch structural examination | 2 times/wk for 20 min | UD |
| 81 | M | G | Osteopathic evaluation + usual care: pre-established osteopathic light-touch structural examination on skull, spine, pelvis, abdomen, upper and lower limbs | 2 times/wk for 20 min | UD |
| 58 | C | L | Sham: range of motion activities, light touch, and simulated OMT techniques. | 1 time/wk for 2 wk and 1 time/mo for 4 mo; each session 15 to 30 min | 5 mo |
|  |  | NT | Time control: no intervention | NA |  |
| 92 | C | L | Sham: hand contact, active and passive range of motion, and techniques that simulated OMT using light touch. | 1 time/wk for 3 wk and 1 time/2 wk for 6 wk; each session 15 min | 9 wk |
|  |  | NT | Sham ultrasound: sham ultrasound therapy was applied on an area of approximately 150 to 200 cm^2^ of the lower at a subtherapeutic intensity of 0.1 W/cm2. | 1 time/wk for 3 wk and 1 time/2 wk for 6 wk; each session 10 min |  |
| 57 | M | L | Sham: manually applied forces of substantially decreased magnitude not in key areas of somatic dysfunction. | NA | NA |
| 72 | M | NA | Sham: postural examination and palpation of different parts of the body. | 1 time/wk for 30 min | 6 wk |
| 70 | C | L | Sham: light touch of the skull and sacrum | 1 time/mo | 6 mo |
|  |  | NT | Usual Care | NA |  |
| 83 | M | NA | Sham: flexion/extension active movements of the hip and knee joints. | 1 time for 4 min | SS |
| 42 | M | G | Sham: passive movements of cervical spine. | NA | NA |
| 47 | M | G | Sham: gentle massage. | 1 time/wk | 2 wk |
| 61 | M | G | Sham: hands-on the area of dysfunction without applying any technique. | 2 or 3 times/wk for 20-30 min | 2 to 3 wk |
| 67 | M | G | Sham: superficial abdominal massage. Hand movements similar to true osteopathic manipulations. | 1 time/2 wk for 45 min | 3 mo |
| 69 | C | L | Sham + drug stable care: light manual contact. Practitioner’s attention diverted by mind task | 1 time/wk for 2 wk, 1 time/2 wk for 4 wk and 1 time/mo for 3 mo; each session 30 min | 18 wk |
|  |  | NT | Usual Care: drug assumption | NA |  |
| 88 | M | NA | Sham: patient’s cranial rhythmic impulse assessment. Similar amount of time as OMT techniques. | 1 time/wk | 4 wk |
| 33 | C | L | Sham: resting hands on the occipital condyles. | 1 time/wk for 8 min | 1 wk |
|  |  | NT | Time control: no intervention | 1 time/wk for 15 min |  |
| 34 | M | S | Sham: hands on the neck mimicking cervical myofascial release technique but with no pressure. | 1 time for 30 min | SS |
| 52 | M | L | Sham: light touch of the cranium, face, neck, shoulders, thorax ,abdomen, pelvis and legs | 1 time/wk for 45 min | 8 wk |
| 64 | M | NA | Sham: hands on the patient simulating osteopathic holds. | 1 time for 6 min (NP group) and 12 min (LBP group) | SS |
| 65 | M | NA | Sham: hands on the patient simulating osteopathic holds. | 1 time for 3.5 min | SS |
| 40 | M | G | Sham: hands on the patient simulating myofascial osteopathic holds. | 3 times/wk for 40 min | 8 wk |
| 56 | M | NA | Sham: hands on mimicking Spencer technique with no corrective forces | 2 times/wk for 6 wk and 1 time/mo for2 mo; each session 30 min | 14 wk |
| 49 | M | NA | Sham: hands on the subject's neck, avoiding any engagement with palpation feelings | 1 time for 0.5 min | SS |
| 37 | C | L | Sham: resting hands on the occiput and C2-C3. | 1 time/wk for 10 min | 3 wk |
|  |  | NA | Time control: no intervention |  |  |
| 91 | M | L | Sham: light touch applied to the same anatomic regions as in the osteopathic group. | 1 time for 20 min | SS |
| 62 | M | NA | Sham: hands on the infant simulating osteopathic holds | 1 time/wk for 45 to 60 min | 1 mo |
| 27 | M | L | Sham: similar to the intervention without applying any techniques | 1 time | SS |
| 38 | M | NA | Sham: hands on the infant simulating osteopathic holds. | 1 time for 4 min | SS |
| 85 | M | L | Sham = light touch, avoiding focal areas of pressure by using flat, soft hand contact | 4 to 7 times/wk for 15 min | UD |
| 87 | M | L | Sham + placebo echinacea: osteopathic examination only (palpation of the cranial bones and muscles and other structures) without treatment maneuvers. | 1 time/2 wk | 12 wk |
|  |  | L | Sham + real echinacea: osteopathic examination only (palpation of the cranial bones and muscles and other structures) without treatment maneuvers. |  |  |
| 71 | M | L | Sham: light touch (same areas, same duration) as study group engaging subject in conversation. | 1 time | SS |
| 74 | C | L | Sham + usual care: hands on the patient simulating myofascial osteopathic holds | 1 time/day | UD |
|  |  | NT | Usual care | NA |  |
| 60 | M | L | Sham: standardized light touch protocol mirroring the OMT protocol | 1 time for 6 min | SS |
| 73 | M | L | Sham: light pressure of a few ounces of force applied to the cranium without OMT. | 1 time for 5 min | SS |
| 63 | C | L | Sham: pre-established osteopathic light-touch musculoskeletal examination | 1 time/2 wk for 5 to 10 min | 5 mo |
|  |  | NA | Usual care | NA |  |
| 59 | M | L | Sham: light manual contact. Practitioner’s attention diverted by mind task | 1 time for 20 min | SS |
| 46 | M | S | Sham: hands on the patient simulating osteopathic holds. | 1 time | SS |
| 50 | M | S | Sham: hands on the lower right paravertebral region without applying any pressure | 3 times/wk for 6 min | 1 wk |
| 53 | M | S | Sham: hands on the patient simulating osteopathic holds. | 1 time | SS |
| 86 | M | NA | Sham: hands on the patient simulating osteopathic thrust manipulation holds. | 1 time | SS |
| 36 | C | L | Sham: series of static bilateral light touch hand placements on the posterior C7/T1 region with verbal reinforcement | 1 time for 1 min | SS |
|  |  | NT | Sham ultrasound: switched off ultrasound with verbal reinforcement |  |  |
|  |  | NT | Time control: no intervention |  |  |
| 89 | M | NA | Sham: passive movement. | 1 time for 5 min | SS |
| 44 | M | NA | Sham: force applied to an adjacent restricted joint. | 1 time/wk for 30 min | 10 wk |
| 55 | M | NA | Sham: hands on the patient simulating osteopathic holds. | 1 time for 20 min | SS |
| 35 | C | H | Sham: side-lying lumbar roll setup without follow through | 1 time | SS |
|  |  | NT | Time control: no intervention |  |  |
| 51 | M | T | Sham: hands on the patient simulating osteopathic holds using. | 1 time | SS |
| 41 | M | NA | Two fingers on the mastoid processes avoiding palpation feeling engagement | 1 time | SS |
| 31 | M | G | Gentle manual pressure applied to the region of the thoracic outlet, occipitoatloid, cervicothoracic junctions, and the epigastric region. Upper extremities circumducted at the shoulder through a partial range of passive motion. | 1 time | SS |
| 68 | M | NA | Hands on lumbar and abdomen bodily | 1 time for 5 min | SS |
| 39 | M | NA | Sham: passive movements without reaching the patient's range of motion limit | 1 time for 30 min | SS |
| 84 | M | NA | Sham: fingers on C4 and head rotated by 30°. Position held for 90s then back | 1 time for 90s | SS |
| 75 | N | NT | Sham magnetotherapy: disconnected magnet therapy on cervical and lumbar regions | 2 times/wk for 60 min | 20 wk |
| 45 | N | NT | Sham magnetotherapy | 1 time | SS |
| 32 | N | NT | Sham ultrasound and pleasant conversation | 1 time for 20 min | SS |
| 54 | N | NT | Subtherapeutic ultrasound | 1 time for 20 min | SS |
|  |  | NT | Time control: no intervention |  |  |
| 77 | C | NA | Sham ultrasound + manual contact: tactile and manual stimulation on OMT regions in combination of sham ultrasound. | 1 time/2 wk for 20 min | 14 wk |
|  |  | NT | Usual care |  |  |
| 79 | C | NA | Therapeutic ultrasound + osteopathic evaluation: pre-established osteopathic light-touch musculoskeletal examination in combination with switched off ultrasound. | 1 time/wk for 12 min | 10 wk |
| 90 | N | NT | Sham ultrasound: nonfunctional ultrasound therapy applying sufficient pressure for tactile stimulation in the same anatomical areas of OMT. | 1 time/2 wk for 6 wk and 1 time/wk for 3 wk; each session for 30 min | 9 wk |
|  |  | NT | Usual care | NA | NA |
| 48 | N | NT | Non-operational laser acupuncture with verbal reinforcement | 1 time for 30 sec | SS |
| 23 | N | NT | Detuned short-wave diathermy with machine switched on producing electrical noise and display light | 3 times/wk | 4 wk |
| 66 | N | NT | NSAID placebo drugs: capsules containing either cornstarch or flour. | 14 times/wk | 3 wk |

Wk = week; mo = month; SS = single session; UD = until discharge; M = manual; N = non-manual; C = combined; OMT = osteopathic manipulative treatment; NSAID = non-steroidal antinflammatory drugs; L = light; S = soft; G = gentle; H = hard; T = therapeutic; NT = no touch; NA = not available;

Appendix table 3. Description of the risk of bias scores.

|  | GRADE | Sequence generation | Allocation concealment | Blinding to participants and personnel | Blinding to outcome assessment | Incomplete outcome data | Selective outcome reporting | Reliable and valid tools | Contamination | Adherence to protocol |
| --- | --- | --- | --- | --- | --- | --- | --- | --- | --- | --- |
| Cardoso_ 2015 | low | unclear | unclear | unclear | low | low | low | low | unclear | high |
| Ruffini_2015 | high | low | low | low | low | low | low | low | unclear | high |
| Haller_2015 | high | low | low | low | low | low | low | low | unclear | unclear |
| Cerritelli_2013 | high | low | low | low | low | low | low | low | unclear | unclear |
| Pizzolorusso_2014 | high | low | low | low | unclear | low | low | low | unclear | unclear |
| Cerritelli_2015a | high | low | low | low | unclear | low | low | low | unclear | low |
| Licciardone_2003 | high | low | low | low | low | unclear | unclear | low | unclear | unclear |
| Licciardone_2004 | moderate | unclear | low | unclear | low | unclear | unclear | low | unclear | unclear |
| Licciardone_2013 | high | low | low | low | unclear | low | low | low | low | low |
| Papa_2012 | moderate | low | unclear | unclear | unclear | unclear | unclear | low | unclear | unclear |
| Hubert_2014 | high | low | low | low | unclear | low | low | low | unclear | low |
| Antolinos-Campillo_2014 | high | low | low | low | low | low | low | low | low | unclear |
| Burns_2006 | high | low | low | unclear | unclear | low | low | low | low | unclear |
| Florance_2012 | high | low | low | low | unclear | low | low | low | low | unclear |
| Nourbakhsh_2008 | moderate | unclear | low | low | low | unclear | unclear | low | unclear | unclear |
| Attali_2013 | high | low | low | unclear | low | low | low | low | low | unclear |
| Cerritelli_2015b | high | low | low | low | low | low | low | low | low | unclear |
| Rolle_2014 | low | low | unclear | high | unclear | low | low | low | unclear | unclear |
| Haller_2014 | high | low | low | low | low | low | low | low | low | low |
| Tozzi_2011 | moderate | unclear | unclear | low | unclear | low | low | unclear | unclear | unclear |
| Tozzi_2012 | low | high | unclear | unclear | unclear | low | low | unclear | unclear | unclear |
| Ajimsha_2014 | moderate | unclear | unclear | low | unclear | low | low | low | unclear | unclear |
| Knebl_2015 | moderate | unclear | unclear | low | unclear | unclear | unclear | unclear | unclear | unclear |
| Fryer_2004 | high | low | low | low | low | low | low | low | low | unclear |
| Noll_2008 | low | unclear | unclear | low | unclear | low | low | low | unclear | unclear |
| Philippi_2006 | low | unclear | low | low | unclear | low | low | unclear | low | unclear |
| Sergueff_2002 | low | unclear | unclear | unclear | unclear | uncler | uncler | uncler | uncler | uncler |
| Swender_2014 | low | low | unclear | unclear | unclear | low | low | low | low | unclear |
| Wahl_2008 | high | low | low | low | low | low | low | low | low | low |
| Noll_2013 | low | low | low | low | unclear | high | high | low | unclear | unclear |
| Wieting_2013 | high | low | low | low | low | low | low | low | unclear | unclear |
| Noll_2010 | high | unclear | low | low | unclear | low | low | low | unclear | unclear |
| Sandhouse_2010 | high | low | unclear | low | low | low | low | low | low | unclear |
| Snider_2012 | high | unclear | low | low | unclear | low | low | low | unclear | unclear |
| McPartland_2005 | low | unclear | low | unclear | low | low | unclear | low | unclear | unclear |
| Da Silva_2013 | low | unclear | unclear | unclear | unclear | low | low | low | unclear | unclear |
| Goldstein_2005 | low | low | unclear | unclear | unclear | low | low | low | low | unclear |
| Hamilton_2007 | high | low | low | low | low | low | low | low | low | unclear |
| Mansilla-Ferragut_2009 | low | unclear | low | unclear | low | unclear | unclear | low | unclear | unclear |
| Brose_2013 | high | low | low | unclear | unclear | low | low | low | low | unclear |
| Cleary_1994 | low | low | low | unclear | unclear | low | low | unclear | unclear | unclear |
| Howell_2006 | low | unclear | unclear | unclear | unclear | low | low | low | low | unclear |
| Guiney_2005 | low | low | unclear | unclear | unclear | low | low | low | low | unclear |
| Barnes_2013 | very low | unclear | unclear | unclear | unclear | high | unclear | unclear | unclear | unclear |
| Boët_2013 | high | low | low | low | unclear | low | low | low | unclear | unclear |
| Klein_2013 | high | low | low | low | unclear | low | low | low | unclear | unclear |
| Castro-Sanchez_2011 | high | low | low | high | low | low | low | low | unclear | unclear |
| Curtis_2011 | moderate | unclear | unclear | low | unclear | low | low | low | low | unclear |
| Hensel_2013 | low | unclear | low | low | unclear | high | high | low | unclear | unclear |
| Hensel_2015 | high | low | low | low | unclear | low | low | low | low | unclear |
| Schwerla_2008 | moderate | low | high | low | unclear | low | low | high | low | unclear |
| Licciardone_2010 | high | low | low | low | unclear | low | low | low | low | low |
| Fryer_2004 | high | low | low | low | low | low | low | low | low | unclear |
| Gibson_1985 | high | low | low | unclear | low | low | low | low | low | unclear |
| Wynne_2006 | low | unclear | unclear | unclear | unclear | unclear | unclear | unclear | unclear | unclear |
